# Analysis of Conformational B-Cell Epitopes in the Antibody-Antigen Complex Using the Depth Function and the Convex Hull

**DOI:** 10.1371/journal.pone.0134835

**Published:** 2015-08-05

**Authors:** Wei Zheng, Jishou Ruan, Gang Hu, Kui Wang, Michelle Hanlon, Jianzhao Gao

**Affiliations:** 1 School of Mathematical Sciences and LPMC, Nankai University, Tianjin, People’s Republic of China; 2 State Key Laboratory of Medicinal Chemical Biology, Nankai University, Tianjin, People’s Republic of China; 3 Department of Physical Sciences, Grant MacEwan University, Alberta, Canada; Vrije Universiteit Brussel, BELGIUM

## Abstract

The prediction of conformational b-cell epitopes plays an important role in immunoinformatics. Several computational methods are proposed on the basis of discrimination determined by the solvent-accessible surface between epitopes and non-epitopes, but the performance of existing methods is far from satisfying. In this paper, depth functions and the k-th surface convex hull are used to analyze epitopes and exposed non-epitopes. On each layer of the protein, we compute relative solvent accessibility and four different types of depth functions, i.e., Chakravarty depth, DPX, half-sphere exposure and half space depth, to analyze the location of epitopes on different layers of the proteins. We found that conformational b-cell epitopes are rich in charged residues Asp, Glu, Lys, Arg, His; aliphatic residues Gly, Pro; non-charged residues Asn, Gln; and aromatic residue Tyr. Conformational b-cell epitopes are rich in coils. Conservation of epitopes is not significantly lower than that of exposed non-epitopes. The average depths (obtained by four methods) for epitopes are significantly lower than that of non-epitopes on the surface using the Wilcoxon rank sum test. Epitopes are more likely to be located in the outer layer of the convex hull of a protein. On the benchmark dataset, the cumulate 10th convex hull covers 84.6% of exposed residues on the protein surface area, and nearly 95% of epitope sites. These findings may be helpful in building a predictor for epitopes.

## Introduction

Epitopes are binding areas on antigens. The prediction of b-cell epitopes is critical for the development of vaccines and immunotherapeutic drugs [1]. B-cell epitopes are categorized into linear and conformational epitopes. A linear b-cell epitope is a contiguous amino acid segment in an antigen. A conformational b-cell epitope is located in close proximity in the protein 3-dimensional structure but discontinuous in the protein sequence. The majority of b-cell epitopes are conformational [2].

It is time-consuming and expensive to use experimental techniques to identify b-cell epitopes [3], especially on a genomic scale. Many computational methods have been developed for b-cell epitope prediction [4]. The prediction of linear b-cell epitopes from antigen sequences can be traced back to the 1980s. The first generation of prediction methods is the propensity model. These models utilized a single propensity of the amino acid [2, 5–10], or combined multiple physicochemical propensities to predict epitopes [10–14]. More complicated models were then used to predict epitopes including the neural network [15], hidden Markov model [16], Naïve Bayes model [17] and support vector machine [18–25].

Some other methods use protein structure to predict epitopes, building the model using simple scoring-based approaches. CEP [26] utilizes solvent accessibility to score amino acid surfaces. DiscoTope [27] utilizes surface/solvent accessibility, contact numbers, and amino acid propensity scores. SEPPA [28] combines propensity scores that were based on solvent accessibility and the packing density of amino acids. PEPITO [29] fuses amino acid propensity scores and solvent accessibility, quantified by using half-sphere exposure in a linear regression. EPSVR [30] takes epitope propensity scores, contact numbers, secondary structure composition, conservation, side chain energy surfaces and planarity scores as inputs, and a support vector regression was built. Zhang et al. [31] utilizes the random forest model, while Liu and Hu [32] uses logistic regression with B-factors and the relative accessible surface area as inputs. Bepar is an association patterns model [33]. EPMeta is a consensus model [30]. Epitopia [34, 35] utilizes the Naïve Bayes model, fusing physicochemical and structural-geometrical properties from a surface patch.

Although many methods are proposed, the performance of b-cell epitope predictors is moderate. With the increase of antigen-antibody crystal structures, it is possible to analyze these complex structures. A more detailed description of the b-cell epitope area becomes important. In this paper, we applied four types of depth functions; half-sphere exposure (HSE) [36], Chakravarty depth [37], DPX [38–40], and half-space depth (HSD) [41] to analyze the location of epitopes. Compared with solvent accessibility, depth functions distinguish between atoms just below the protein surface and those in the core of the protein [37–40]. The goal of this paper is to investigate these depth functions and the convex hull to distinguish conformational epitopes from non-epitopes. This information may provide useful clues for b-cell epitope prediction.

## Materials and Methods

### Dataset

The dataset for this paper was first used as benchmark dataset in Ansari and Raghava [42]. It contains 161 protein chains from 144 antigen-antibody complex structures. Sequence redundancy was removed by BLASTCLUST, at 40% cutoff, leaving 57 antigen chains remaining. In this paper, all exposed residues (relative solvent accessibility RSA>0) are considered. The dataset of 57 antigens contains 915 conformational epitopes and 9632 exposed non-epitopes (in S1 Table). In the following section, the term non-epitopes will refer to exposed non-epitopes, i.e. non-epitopes on the antigen protein surface.

### Computed Features

#### RSA

Solvent accessible surface area (ASA) is calculated by NACCESS [43]. Relative solvent accessibility (RSA) is defined as the ratio of the ASA of a residue, observed in its three-dimensional structure, to that observed in an extended tri-peptide conformation. We found that the RSAs of all epitopes are positive, though some values are only slightly larger than zero. For example, the epitope site Val206 of paracoccus denitrificans two-subunit cytochrome C oxidase complex (PDB ID:1AR1:B) has an RSA value of 0.008. To avoid losing any epitope sites, a residue is considered to be an exposed residue if the RSA is greater than 0.

#### Conservation

Conservation measure, our use of which was motivated by Valdar’s 2002 work[44], is defined as follows,

$$\text{Conservation}=1-(-\sum_{k=1..20}\left\{{\text{p}}_{\text{k}}*{\text{log}}_{2}\left({\text{p}}_{\text{k}}\right)\right\}/{\text{log}}_{2}\left(20\right))$$ (1)

where *p*_*k*_ is the value from the Weighted Observation Percentage (WOP) matrix generated by PSI-BLAST [45], divided by 100. If all WOP values of a given residue equal zero, i.e. *p*_*1*_, *p*_*2*_,*…*,*p*_*20*_ is represented by 20 zeroes, then *conservation* is one.

#### Half-Sphere Exposure

Half-Sphere Exposure (HSE) is a 2D measure, introduced by Hamelryck [36]. HSE consists of the number of C_α_ atoms in two half-spheres around a residue’s C_α_ atom. One of the half-spheres corresponds to the side chain’s neighborhood, the other half-sphere is in the opposite direction. There are two ways to compute HSE, depending on whether information is available about both the C_α_and C_β_positions (HSEB) or only about the C_α_positions (HSEA). HSE can be divided into HSEAU, HSEAD, HSEBU and HSEBD, depending on whether the half-sphere selected is an up half-sphere (U) or a down half-sphere (D). In this paper, we calculated HSEAU, HSEAD, HSEBU and HSEBD, with radius 13Å.

#### Chakravarty Depth

Chakravarty [37] defined the depth of an atom in a protein as the distance between the atom and the nearest surface water molecule. The residue depth is the average of the constituent atom depths. Residue depth is calculated by the program *depth-1*.*0* [46].

#### DPX

Atom depth (DPX), first introduced by Pintar [38–40], is defined as the distance between a non-hydrogen atom and its closest solvent-accessible protein neighbor. DPX is a good geometrical descriptor of the protein interior. Residue DPX is the average of the constituent atom DPX values. We calculated residue DPX using the software DPX [38].

#### Half Space Depth (HSD)

Tukey [41] introduced half space depth (HSD) to order the high dimensional data. It is defined as:

$$\text{HSD}\left(\text{x;P}\right)=\text{inf}\{\text{P}\left(\text{H}\right):\;\text{H is closed half space},\;\text{x}\in \text{H},\;{\text{x in R}}^{\text{d}}\}$$ (2)

where *x* is a point in d-dimensional space with probability measure *P*. HSD is defined as the minimum probability mass carried by any closed half space containing *x*. For a protein, the probability P(H) is estimated by the empirical distribution. The i-th residue HSD is then defined by:

$$\text{HSD}\left({\text{Res}}_{\text{i}}\right)=\text{inf}\left\{\#\left({\text{plane}}_{\text{Resi}}\right)/\text{N}\right\}$$ (3)

where #(plane_Resi_) is the number of residues in the half space which is divided by the plane through that i-th residue, and N is the total number of residues in the protein. The use of residue HSD is motivated by Shen [47, 48].

#### Convex Hull

In mathematics, the convex hull of a set X of points in Euclidean space is the smallest convex set that contains X. For instance, when X is a bounded subset of the plane, the convex hull may be visualized as the shape formed by a rubber band stretched around X. For a protein, we consider all residues in a protein to be a point set X. In this paper, MIConvexHull (http://miconvexhull.codeplex.com/) is used to calculate the convex hull of a point set. [49].

#### K-th Convex Hull

Let X be all the atoms of the exposed residues (RSA>0) in a protein. Atoms which are on the convex hull surface are defined as the first level of the convex hull, denoted as CH_1_(X). The remaining atoms of the protein comprise the set X-CH_1_(X). We then compute the convex hull of X-CH_1_(X), such that the atoms on the convex hull surface of X-CH_1_(X) are defined as the secondary level of the convex hull of the protein, denoted as CH_2_(X). Generally speaking, the k-th level of the convex hull of the protein is the convex hull of the set X-∪_i = 1..k-1_{CH_i_(X)}, denoted as CH_k_(X). Finally, one protein can represent a union of *n* convex hulls, i.e. X = ∪_i = 1..n_{CH_i_(X)}. The level of the k-th convex hull of a residue is defined as the minimal level of convex hulls of atoms that are in the residue. For instance, Glycine contains C_α_,C_β_, N,O atoms, where C_α_∈CH_2_(X), C_β_∈CH_3_(X), N∈CH_3_(X), and O∈CH_5_(X). The level of the convex hull for Gly is 2, as that is the minimal of {2, 3, 3, 5}. The k-th convex hull is a useful tool for classifying the residues. Take the exposed residues of a protein for example; all exposed residues can be divided into k convex hulls.

#### Cumulate k-th Convex Hull

For given exposed atoms of a protein X, we can calculate the k-th convex hull (CH_k_(X)) for each residue. The cumulate k-th convex hull of X (denoted as CH^k^(X)) is defined as the union of *k* convex hulls, i.e. CH^k^(X) = ∪_i = 1..k_{CH_i_(X)}. Obviously, CH^m^(X) is a strict subset of CH^n^(X), if *m<n*. If a residue is in CH_i_(X), then this residue is also in the cumulate *i*-th convex hull CH^i^(X). Clearly, we can choose a finite value K_0_ for which CH^k^(X) will cover (contain) all the exposed epitopes on the protein surface. K_0_ is defined as follows:

$${\text{K}}_{0}=\text{min}\{\text{k}|\;\text{all epitopes}\in {\text{CH}}^{\text{k}}\}$$ (4)

K_0_ would be different for different antigen proteins. If there are more than two chains in the antibody-antigen complex, the exposed atoms of all chains will be used to compute the convex hull and the cumulate convex hull. We developed software for calculating the Convex Hull of Protein Surface (CHOPS), which is available at <www.sourceforge.net/projects/chops>.

**Fig 1:**
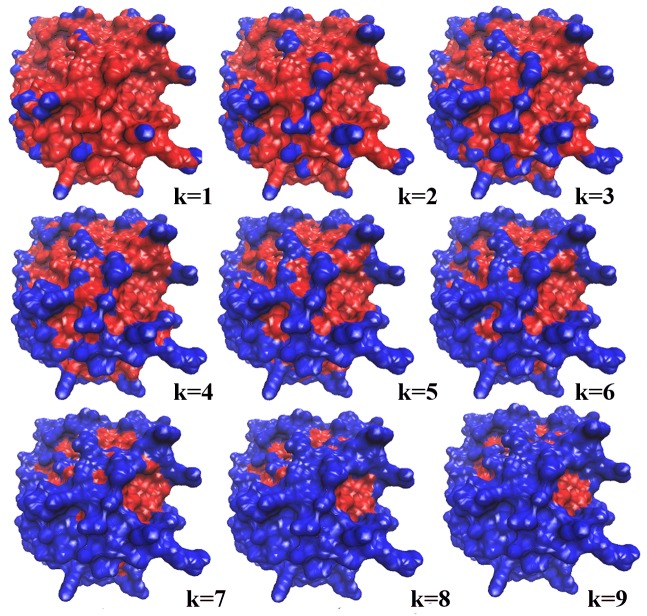
The cumulate k-th convex hull of the protein 1NCA:N. (k = 1, 2,…9). Atoms on the cumulate k-th convex hull are colored blue, remaining exposed atoms are colored red.

The cumulate k-th convex hull of protein 1NCA:N is shown in Fig 1, where k = 1, 2,…, 9. Atoms on the cumulate k-th convex hull are colored blue, and the remaining exposed atoms are colored red. The proportion of exposed atoms on the convex hull to exposed atoms is increased with increasing value of k.

#### Statistical Features on the K-Th Convex Hull

The coverage ratio of *epitopes* in the k-th convex hull (CREPI_k_) of a protein is the number of epitopes in the k-th convex hull divided by the total number of *epitopes* in this protein. The coverage ratio of *epitopes* in the *cumulate* k-th convex hull (CREPI^k^) is the number of epitopes in the cumulate k-th convex hull divided by the total number of *epitopes* in the protein. The coverage ratio of exposed residues in the k-th convex hull (CREXP_k_) is the number of *exposed residues* in the k-th convex hull divided by the number of total *exposed residues* in the protein. The coverage ratio of exposed residues in the cumulate k-th convex hull (CREXP^k^) is the number of *exposed residues* in the *cumulate* k-th convex hull divided by the number of total *exposed residues* in the protein. The proportion of epitopes in the k-th convex hull (PROP_k_) is defined as the number of *epitopes* in the k-th convex hull divided by the number of *all residues* in the k-th convex hull. The proportion of epitopes in the cumulate k-th convex hull (PROP^k^) is defined as the number of *epitopes* in the cumulate k-th convex hull divided by the number of *all residues* in the cumulate k-th convex hull.

For example, there are 10 epitopes and 200 exposed residues on an antigen protein. Two epitopes and 20 residues are in the first convex hull. Then, CREPI_1_ is 2/10 = 20%, CREXP_1_ is 20/200 = 10%, and PROP_1_ is 2/20 = 10%.

## Results and Discussion

### Amino Acid Composition of Epitopes and Non-Epitopes

**Fig 2:**
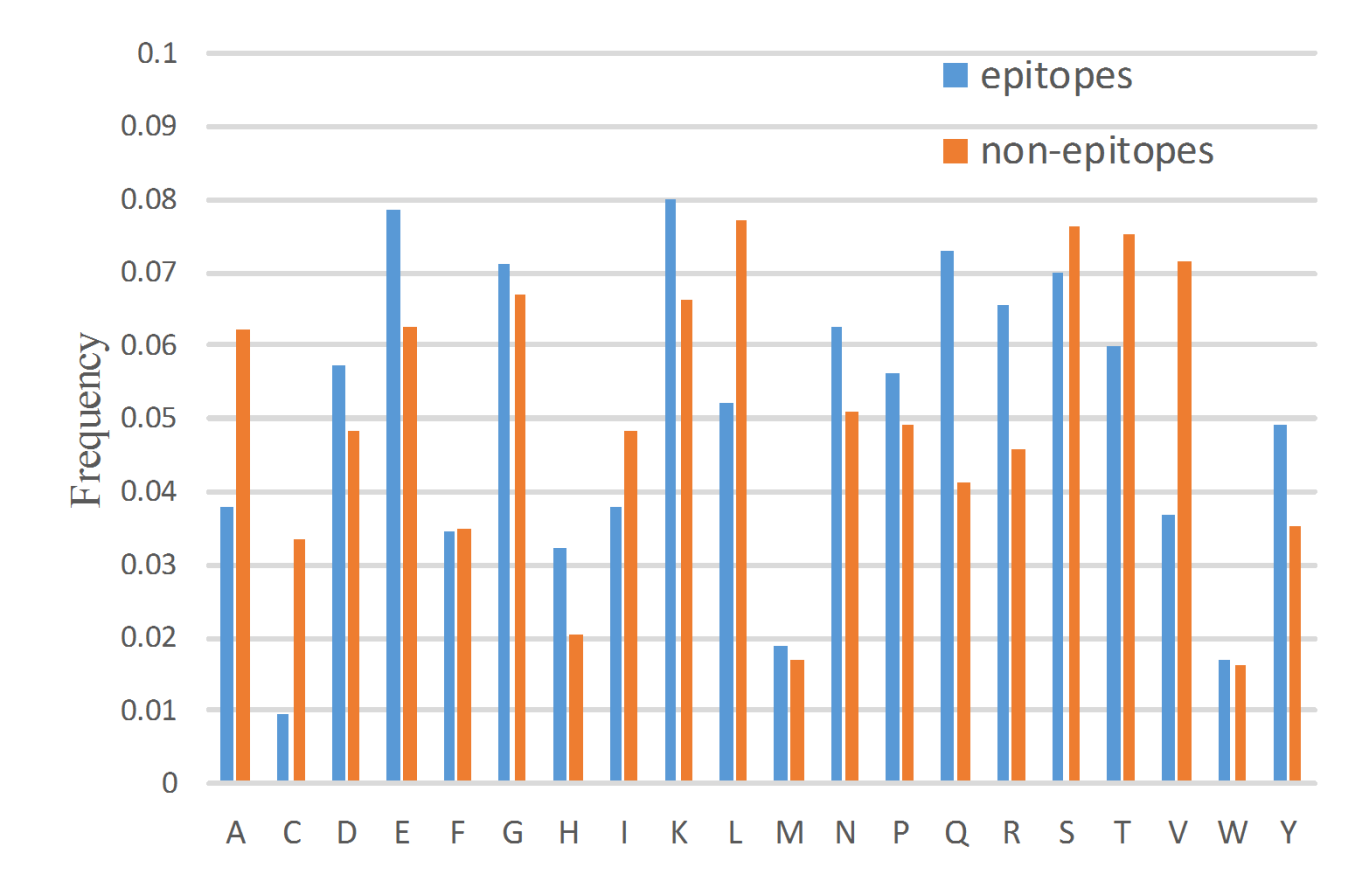
Amino acid composition of epitopes and non-epitopes.

All exposed residues (RSA>0) are considered. In the following sections, the term non-epitopes will refer to non-epitopes with RSA values larger than zero. Amino acid composition is defined as the count of a type of amino acid divided by the length of the antigen protein. Fig 2 shows the average amino acid composition for 57 antigens. It shows that conformational b-cell epitopes are rich in negatively charged residues Asp(D) and Glu(E); positively charged residues Lys(K), Arg(R), and His(H); non-polar, aliphatic residues Gly(G) and Pro(P); polar, non-charged residues Asn(N) and Gln(Q), and the aromatic residue Tyr(Y). Compared to the epitopes, non-epitopes are rich in non-polar, aliphatic residues Ala(A), Leu(L), and Val(V), and the polar, non-charged residue Ser(S). These results are consistent with previous findings that epitopes are rich in polar amino acids and aromatic amino acids but depleted in aliphatic amino acids [33, 50, 51].

### Secondary Structure of Epitopes and Non-Epitopes

**Fig 3:**
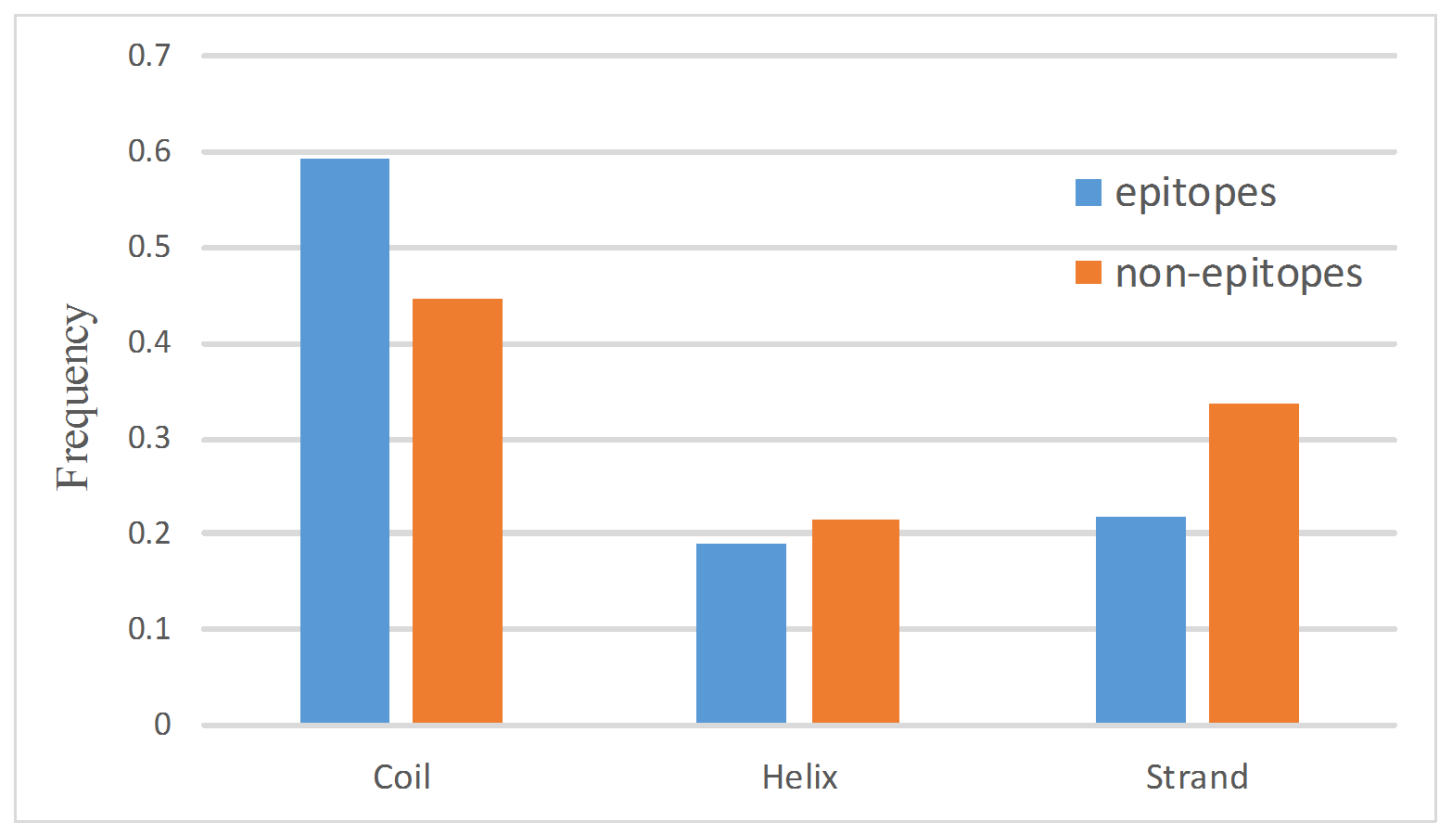
Secondary structures of epitopes and non-epitopes.

Secondary structure was computed by the DSSP program, and then eight types of secondary structure are combined into three types: (1) Helices, which groups α-helices, 3-helices and π-helices. (2) Strands, that is, isolated β−bridges and extended strands participate inβ−ladders. (3) Coils, consisting of hydrogen-bonded turns, bends and others. The secondary structures of epitopes and non-epitopes are shown in Fig 3. Conformational b-cell epitopes are rich in coils. In contrast, the non-epitopes are rich in strands and helices. Further analyzing the eight types of secondary structure from DSSP, we see that epitopes are rich in bends (S) and hydrogen-bonded turns (T). In contrast, non-epitopes are rich in extended strands which participate inβ−ladders (E), andα-helices (H). (See S1 Fig).

### RSA Values of Epitopes and Non-Epitopes

**Fig 4:**
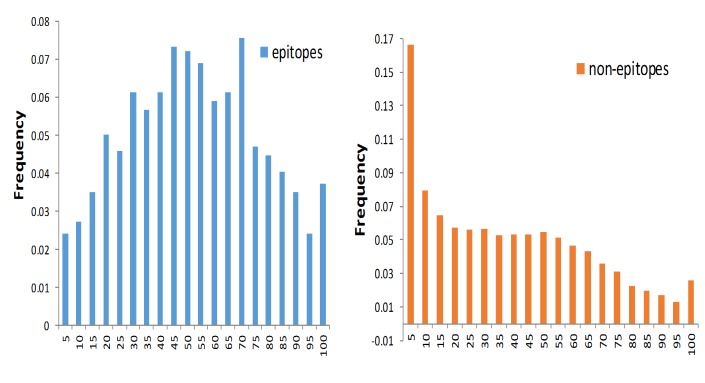
Histogram of RSA values for epitopes and non-epitopes. (A) RSA values of epitopes (red) (B) RSA values of non-epitopes (blue).

Fig 4 shows the histogram of RSA values of both epitopes and non-epitopes. Fig 4A shows the histogram for epitopes alone. The average and standard deviation of the RSA values of epitopes are 50.0 and 24.8, respectively (see Table 1). Fig 4B shows the histogram for non-epitopes alone. The average and standard deviation of the RSA values are 35.4 and 27.4, respectively. The distributions for epitopes and non-epitopes do not follow the normal distribution, based on the Shapiro-Wilk normality test. The average RSA of epitopes is significantly greater than the average RSA of non-epitopes, based on the Wilcoxon rank-sum test (p-value<2.2e-16).

**Table 1:** Average values and standard deviation (in bracket) of depth function for epitopes and non-epitopes.

Depth	Epitopes	Non-epitopes	PCC between depth and RSA	Significance difference between epitopes and non-epitopes (p-value<0.05)
Chakravarty depth^a^	4.15(0.69)	5.05(1.79)	-0.78(0.04)	-^b^ (p-value< 2.2e-16)
DPX^c^	0.41(0.41)	0.67(0.62)	-0.70(0.04)	- (p-value< 2.2e-16)
HSEAU^d^	8.46(6.76)	13.1(9.1)	-0.78(0.08)	- (p-value< 2.2e-16)
HSEAD ^d^	17.97(6.55)	19.2(7.3)	-0.01(0.14)	- (p-value = 2.8e-06)
HSEBU ^d^	9.64(6.83)	14.2(8.7)	-0.79(0.04)	- (p-value< 2.2e-16)
HSEBD ^d^	17.11(6.51)	18.4(7.3)	-0.07(0.14)	- (p-value = 1.0e-06)
HSD^e^	0.02(0.04)	0.05(0.07)	-0.55(0.13)	- (p-value< 2.2e-16)
RSA	50.0(24.8)	35.4(27.4)	1.00	+ (p-value< 2.2e-16)

^a^Chakravarty depth is introduced by [37] and computed following [46].

^b^ means that the average of conservation scores is significantly smaller than the average of conservation scores of non-epitopes at p-value<0.05 using Wilcoxon rank sum test with one-side test.

^c^ DPX is the average of constituent atom DPX values, DPX is calculated following [38].

^d^HSEAU/HSEAD: Half-Sphere Exposure using C_α_ position and selected Up/Down half-sphere, HSEBU/HSEBD: Half-Sphere Exposure using C_β_ position and selected Up/Down half sphere. Radius of sphere 13Å is used.

^e^HSD: half space depth which was firstly introduced by [41], and used in [47, 48].

Correlations between RSA and depth functions are also considered. Table 1 shows average 57 antigen proteins Pearson correlation coefficient (PCC) between RSA and depth. Chakravarty depth, DPX, HSEAU and HSEBU obtain higher correlation with absolute PCC of (> 0.70). HSEAD, HSEBD and HSD obtain lower correlation with absolute PCC of (<0.55). Half-sphere exposure using up half-sphere (HSEAU, HSEBU) achieves higher correlation coefficient than the using down half-sphere (HSEAD, HSEBD).

### Depth Functions for Epitopes and Non-Epitopes

Table 1 shows the average values of different depth functions for epitopes and non-epitopes. The average epitope depth values, i.e. the Chakravarty depth, DPX, Half Space Exposure (HSE) and Half Space Depth (HSD), are smaller than the average non-epitope depth values. Take the Chakravarty depth for example; the average depth for epitopes is 4.15, which is lower than average depth for non-epitopes, 5.05. This indicates that epitopes prefer the outer layer of the protein surface, making it easier for epitopes to interact with antibodies.

### Conservation, Depth Functions, and RSA for Epitopes and Non-Epitopes

**Fig 5:**
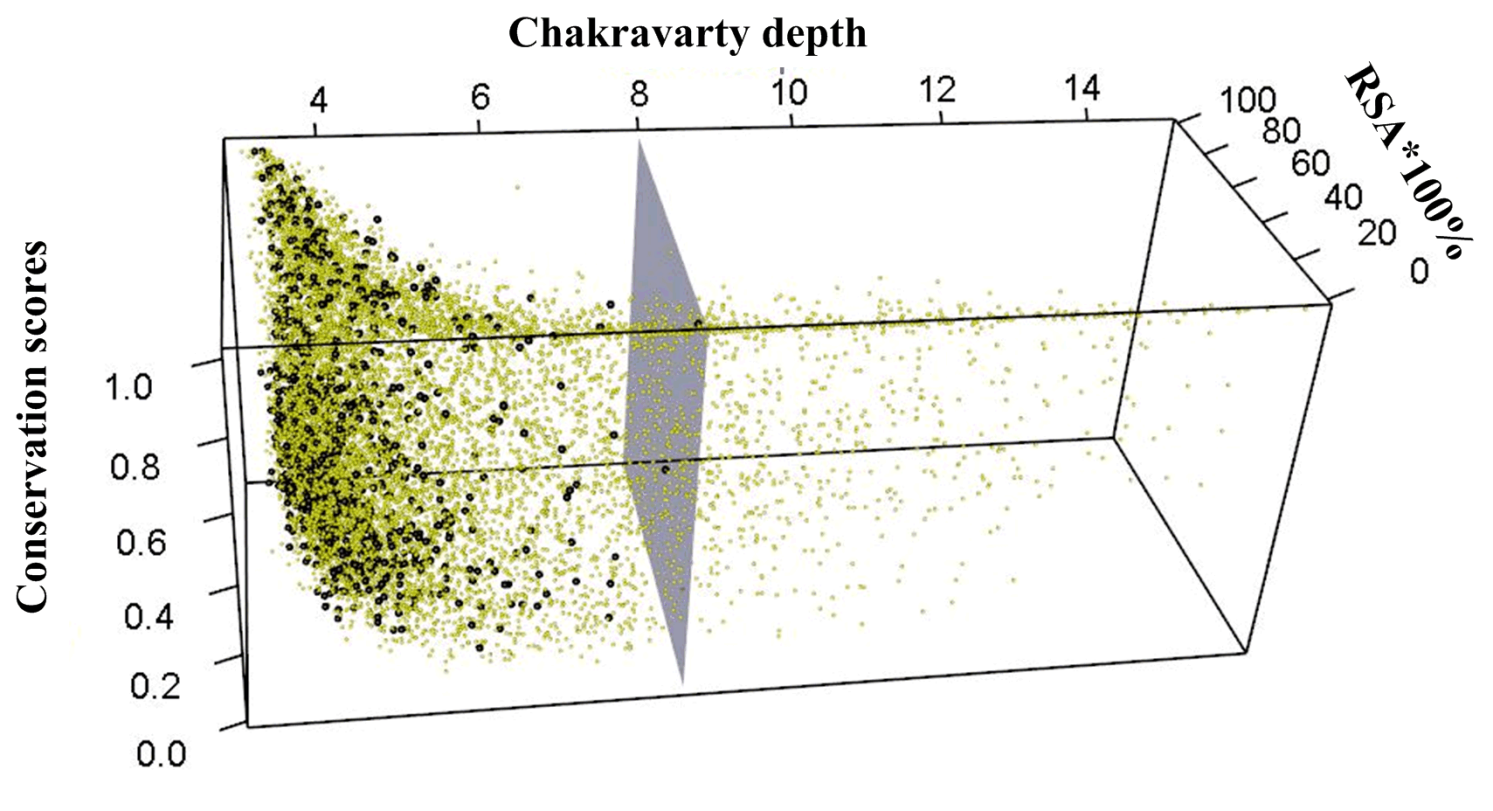
Chakravarty depth, RSA and conservation of epitopes (black points) and non-epitopes (yellow points). Depths of all epitopes are less than 8Å (gray plane).

The conservation of epitopes is widely used in the prediction of epitopes. The average conservation scores for epitopes are not significantly lower than for non-epitopes. We further analyze the relationship between RSA, Chakravarty depth and conservation (Fig **5**). It shows that Chakravarty depth for all epitopes is below 8Å. Further examination of non-epitopes with residue depth above 8Å shows that the median RSA and the conservation of these residues are 1.4 and 0.84, respectively, In contrast, the median of the RSA and the conservation for epitopes are 49.5 and 0.36. With information about the depth function, the epitopes are easily distinguished from non-epitopes.

### Analysis of Epitopes Using the Convex Hull (CH_k_)

We calculate the level of the convex hull for each epitope. The smaller the level, the more external the layer in which the epitope is found. The average level of the convex hull for epitopes is 4.5, while the non-epitopes are at 8.0. The level of the convex hull is significantly smaller (p-value < 2.2e-16) than for non-epitopes, using the Wilcoxon rank-sum test. This indicates that epitopes are closer to the convex hull. It is also consistent with the results in Rubinstein et al. [51] which found that the distance between epitope site and protein convex residue is less than the distance between non-epitope site and convex residue.

**Fig 6:**
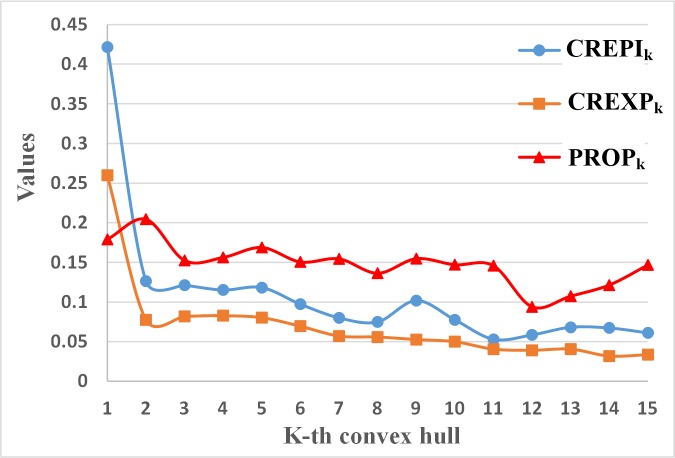
Average CREPI_k_, CREXP_k_ and PROP_k_ in different k-th surface convex hull layers CH_k_. CREPI_k_ is the number of epitopes in the k-th convex hull divided by the total number of *epitopes* in this protein. CREXP_k_ is the number of *exposed residues* in the k-th convex hull divided by the number of total *exposed residues* in the protein. PROP_k_ is defined as the number of *epitopes* in the k-th convex hull divided by the number of *all residues* in the k-th convex hull.

Epitopes prefer the outer layer of the protein surface, but not all epitopes are in the first convex hull (CH_1_(X)) of the protein. Fig 6 shows the average CREPI_k_, CREXP_k_ and PROP_k_ in the k-th surface convex hull, where k = 1,…15. There are 42.2% of the epitopes in 1^st^ convex hull of the protein (CREPI_1_ = 42.2%). These 42.2% of epitopes cover 26.0% of the surface area of the protein (CREXP_1_ = 26.0%). There is approximately one epitope per six residues of CH_1_ (PROP_1_ = 17.9%, 1/0.179≈6). We also noted that CREPI_k_, CREXP_k_ and PROP_k_ are decreased while k is increasing. This indicates that the percentage of epitopes would be decreased when the residue in the protein interior. Take the k = 2 for example; there are 12.6% of the epitopes in the secondary convex hull (CH_2_). These residues in CH_2_ cover 7.76% of the protein surface. Each epitope is around 4 non-epitopes.

**Fig 7:**
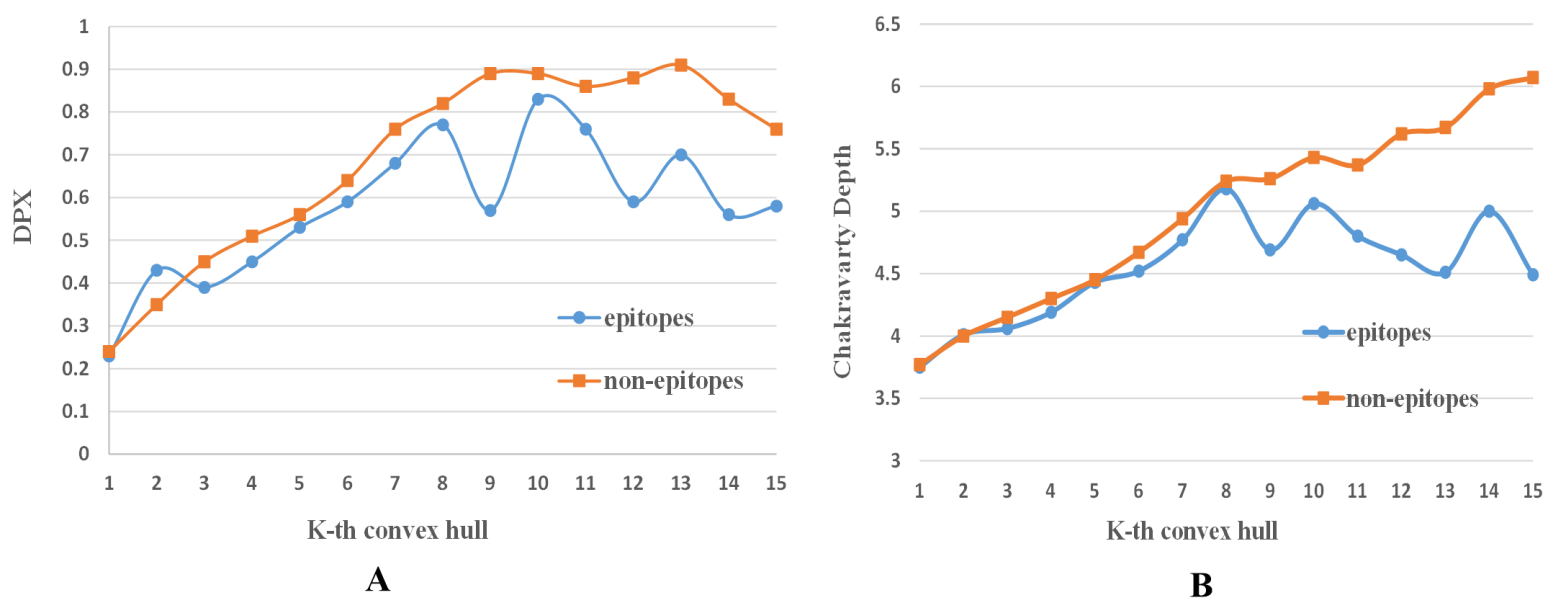
Different depth functions according to the k-th convex hull layers CH_k_ (k = 1, 2,…, 15). (A)DPX (B) Chakravarty Depth.

We also analyzed the influence of the depth function according to the k-th convex hull. Fig 7 shows these results. For DPX (Fig 7A), the values for epitopes are smaller than the values for non-epitopes, except on CH_2_. For Chakravarty depth (Fig 7B), all values for epitopes are smaller than the values for non-epitopes. There are two types of HSE, i.e. HSEA, HSEB. There is no significant rule for the HSEA down sphere (HSEAD) (See S2 Fig), but all values of HSEAU for epitopes are smaller than the values of HSEAU for non-epitopes, except CH_4_. On the other hand, there is no significant rule for the HSEB down sphere (HSEBD), all values of HSEBU for epitopes are smaller than the values of HSEBU for non-epitopes (S3 Fig). This indicates that the DPX, Chakravarty depth and HSEAU, HSEBU may be useful to classify epitopes and non-epitopes on the surface.

### Minimal Level of the Convex Hull for Antigen Proteins

**Fig 8:**
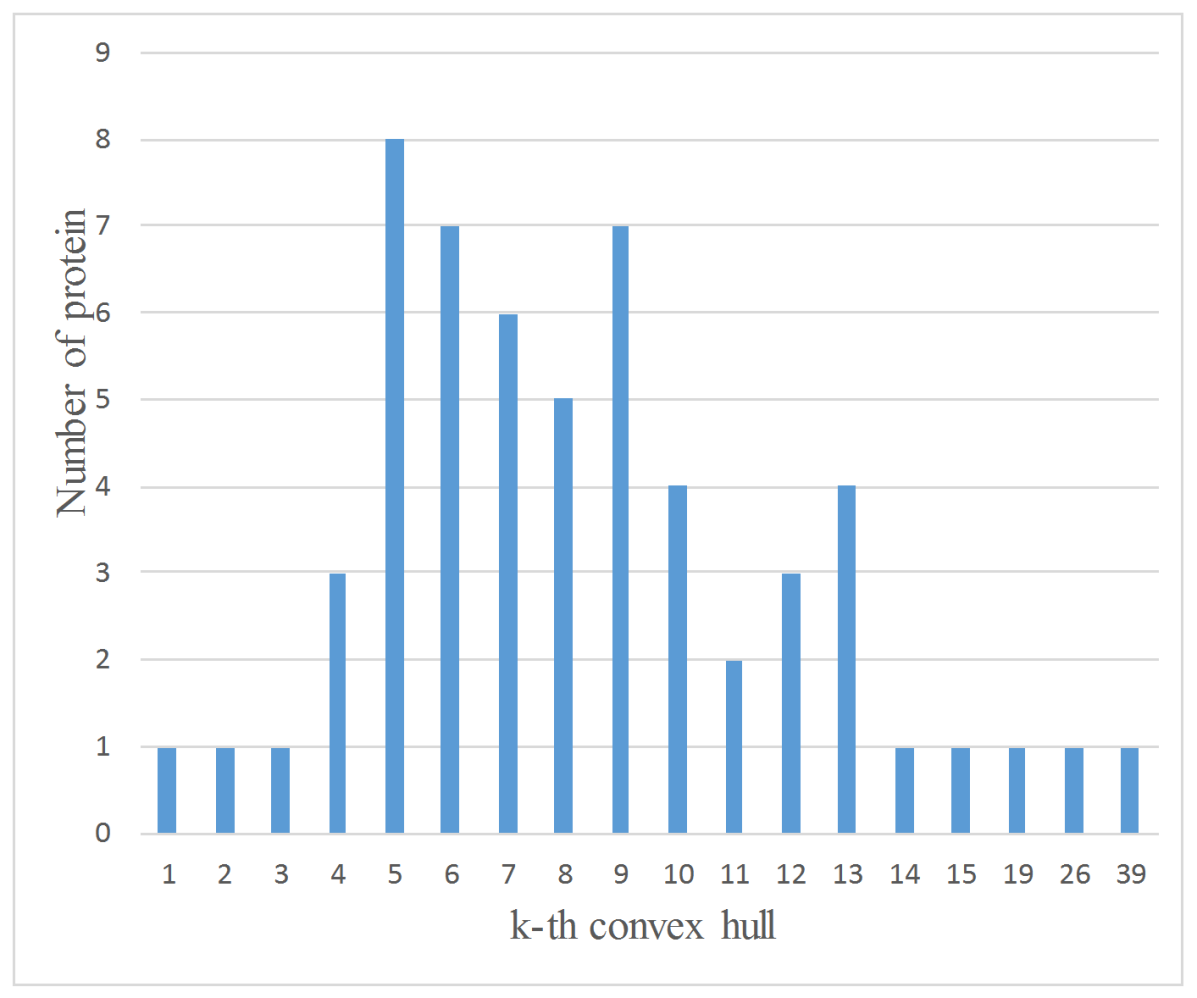
Minimal level of the convex hull for antigen proteins. Take K_0_ = 7 for example, there are six proteins for which all epitopes are located in the cumulate 7-th convex hull (CH^7^).

K_0_ is the minimal level of the convex hull such that all epitopes are on the cumulate k-th convex hull (CH^k^). We analyzed the cumulate k-th convex hull of different antigen protein chains. Fig 8 shows the results. For 24.6% ((1+1+1+3+8)/57, K_0_≤5) of the antigen chains, all epitopes are covered in the top five layers of the convex hull of the antigen. There are a total of 86.0% ((3+8+7+6+5+7+4+2+3+4)/57 = 86.0%) proteins for which all epitopes are located in top 4~13 layers of the convex hull. This also indicated that there is only one protein for which all epitopes are located in the first layer of the convex hull (CH^1^). From these results, we can see that the convex hull functions can further describe the distribution of epitopes.

### Choose K_0_ for the Cumulate K-Th Surface Convex Hull

**Fig 9:**
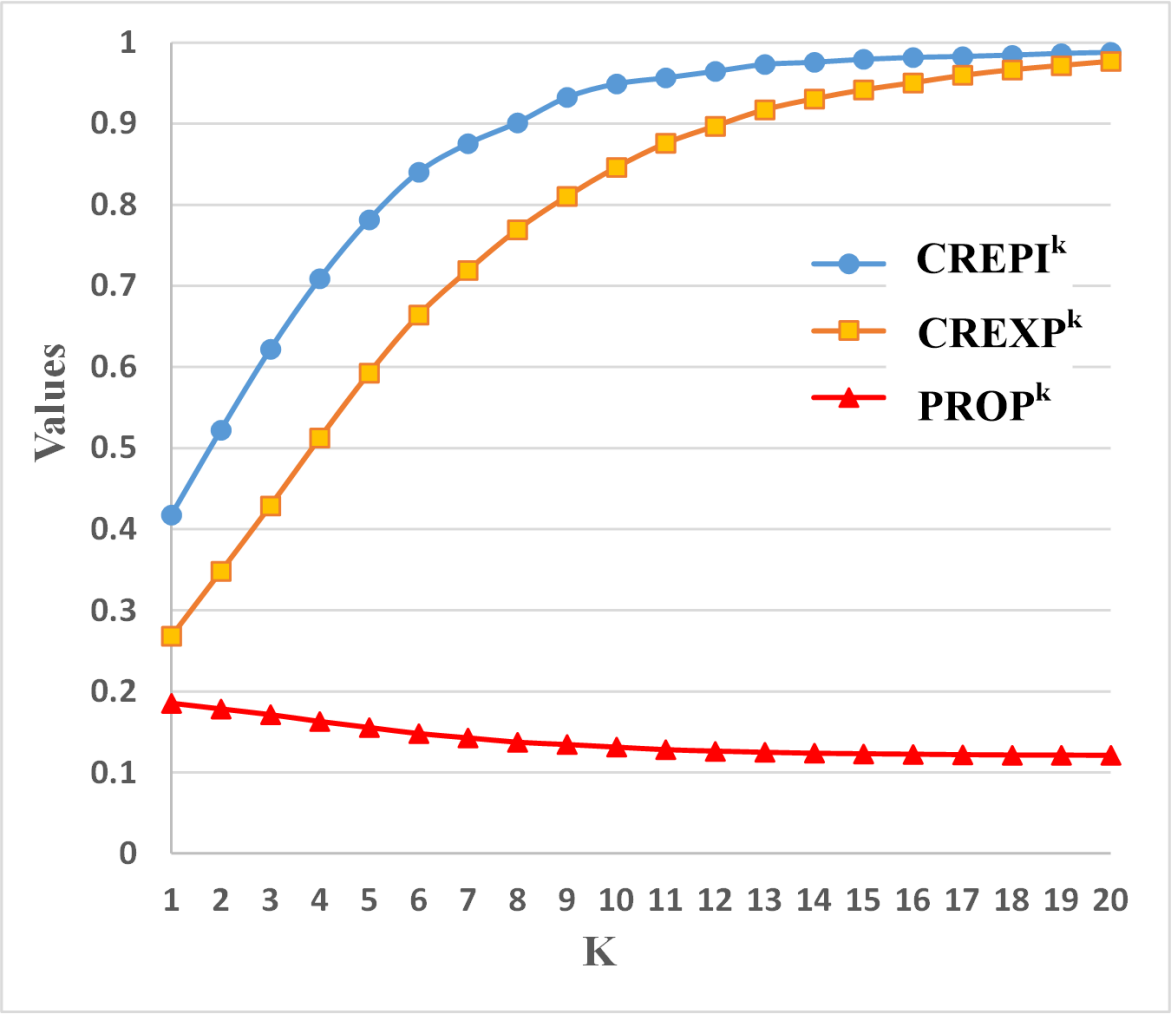
CREPI^k^, CREXP^k^ and PROP^k^ curve of CH^k^ with different k values. CREPI^k^ is the number of epitopes on the cumulate k-th convex hull divided by the total number of *epitopes* in the protein. CREXP^k^ is the number of *exposed residues* on the *cumulate* k-th convex hull divided by the number of total *exposed residues* in the protein. PROP^k^ is defined as the number of *epitopes* in the cumulate k-th surface convex hull divided by the number of *all residues* in the cumulate k-th convex hull.

For a given protein, we do not know which K_0_ of the cumulate k-th convex hull can contain all the epitope sites. If the K_0_ value is too small, many epitopes will not be considered. On the other hand, if the K_0_ value is too big, too many non-epitopes will be considered. To estimate a proper K_0_ value, 7 chains (protein IDs: 1AFV:A, 1FSK:A, 1IAI:M, 1KB5:A, 1NFD:D, 1OTS:A, 1QFU:A) were randomly selected as a test set, and the remaining 50 chains were used as training set. We calculated CREPI^k^, CREXP^k^ and PROP^k^ of CH^k^ (k = 1, 2,…, 20) for each protein in the training set. Then, averages for three kinds of ratios in CH^k^ were computed. Fig 9 shows the results.

As the k value is increased, CH^k^ will contain many more residues, including both epitopes and non-epitopes. The more epitopes included in CH^k^, the larger CREPI^k^ will be. The more non-epitope sites included in CH^k^, the larger CREXP^k^ will be, and the smaller PROP^k^ will be (see Fig 9). If we want to obtain a proper k value, we must make sure CREPI^k^ and PROP^k^ are as large as possible, and CREXP^k^ is as small as possible at the same time. For this training dataset, K_0_ = 10 is selected. Generally, CH^10^ covers 84.6% of the residues on the protein surface area, and nearly 95% of the epitope sites. In CH^10^ of a protein, 13.1% of the residues are epitopes.

We test our results on the test dataset. CREPI^10^, CREXP^10^ and PROP^10^ of CH^10^ for each protein are calculated. The average of CREPI^10^s is 96.7%. The CREPI^10^ values for five proteins are above 95%, except for 1AFV:A (92.8%) and 1QFU:A(84.2%). The average of the CREXP^10^s is 77.3%. This indicates that just 77.3% of the exposed residues are covered in CH^10^. The average value of PROP^10^ is 8.2%. We further analyze CH^6^ for the test dataset, and CH^6^ for 1FSKA, 1IAIM, 1KB5A, 1OTSA covers 100% of the epitopes. The remaining 7^th^, 8^th^, 9^th^, 10^th^ layers of the convex hull contain zero epitopes. This indicates that the cutoff of K_0_ = 10 is probably robust for the test data.

## Conclusions

Relative Solvent Accessibility or solvent surface is widely used in the analysis of proteins and protein functions. The accessible surfaces are always the residues for which RSA >5%. If the cutoff 5% is used, about 2.4% of the epitopes are buried, in the dataset used in this paper. We use the k-th convex hull and the cumulate k-th convex hull to categorize the protein residues, and analyze the location of epitopes on different layers of the proteins. On each layer of the protein, we compute the four different types of depth functions to analyze the location of epitopes on different layers of the proteins.

Based on RSA of the epitopes and non-epitopes on the protein surface, the average RSA for epitopes is significantly greater than the average RSA for non-epitopes. Nevertheless, there is no significantly difference between RSA values of epitopes and non-epitopes which are in top eight convex hull layers (see S4 Fig). It may be the reason why the b-cell prediction performance is moderate using monotonous RSA-based features. For Chakravarty depth, DPX, Half Sphere Exposure, and HSD, the average values for epitopes are significantly lower than the average values for non-epitopes on the surface. Take Chakravarty depth for example; all epitopes have depth below 8Å. This indicates that epitopes may be distinguished from non-epitopes on the basis of the depth function.

Correlation between RSA and different depth functions are also analyzed. Chakravarty depth, DPX, Half-sphere exposure using up half-sphere(HSEAU, HSEBU) achieve higher absolute Person correlation coefficients. HSD, half-sphere exposure using down half-sphere (HSEAD, HSEBD) and half space depth(HSD) achieve lower absolute Person correlation coefficients. Depth functions provide more detailed description of the b-cell epitopes It may provide useful clues for b-cell epitope prediction.

The conservation for epitopes is not significantly lower than that for non-epitopes. This is due to the fact that some non-epitopes may play important biological functions, such as glycosylation sites and pockets, giving higher conservation. For example, for the residue LEU259 of hiv-1 JR-RF gp120 core protein (2B4C:G), its Chakravarty depth is 13Å, RSA is just 0.2, while its conservation is 0.98; this residue is a glycosylation site.

Epitopes prefer to be located in the outer layer of the protein surface, but not all epitopes are in the convex hull of the protein. For Chakravarty depth, HSEAU, and HSEBU, the average depth function values for epitopes are smaller than the average values for non-epitopes on the surface. On the benchmark dataset, CH^10^ just covers 84.6% of the residues on protein surface area, but nearly 95% of the epitope sites.

Our software for calculating the Convex Hull of Protein Surface (CHOPS) can be downloaded from <www.sourceforge.net/projects/chops>. As demonstrated in a series of recent publications [52–63] in developing new prediction or analysis methods, user-friendly and publicly accessible web-servers will significantly enhance their impacts [64]. We shall make efforts in our future work to provide a web-server for the method presented in this paper.

## Supporting Information

S1 Fig Secondary structure of epitopes and non-epitopes. B: residue in isolated β-bridge; E: extended strand, participates in β-ladder; G: 3-helix; H: α-helix; S: bend; T: hydrogen bonded turn; I: 5-helix.
(DOC)

S2 Fig HSEA depth functions according to the k-th convex hull layers CH_k_ (k = 1, 2,…, 15). HSEA:Half-Sphere Exposure using information only about the C_α_ position.
(DOC)

S3 Fig HSEB depth functions according to the k-th convex hull layers CH_k_ (k = 1, 2,…, 15). HSEB: Half-Sphere Exposure using information about both the C_α_ and C_β_ positions.
(DOC)

S4 Fig RSA according to k-th convex hull layers CH_k_ (k = 1, 2,…, 15). There is no significantly difference between epitopes and non-epitopes in the top 8 convex hull layers (p-values are 0.82, 0.08, 0.60, 0.37, 0.53, 0.42, 0.24 and 0.64 using Wilcoxon rank sum test with two sides).
(DOC)

S1 Table RSA and different depth values for epitopes and exposed non-epitopes. (XLSX)
