# Supplementary material for: Analysis of Conformational B-Cell Epitopes in the Antibody-Antigen Complex Using the Depth Function and the Convex Hull
Source: PLoS One. 2015 Aug 5;10(8):e0134835. doi: 10.1371/journal.pone.0134835 (PMC4526569; doi:10.1371/journal.pone.0134835)
Supplement: S1 Fig — B: residue in isolated β-bridge; E: extended strand, participates in β-ladder; G: 3-helix; H: α-helix; S: bend; T: hydrogen bonded turn; I: 5-helix. (DOC) [file pone.0134835.s001.doc]

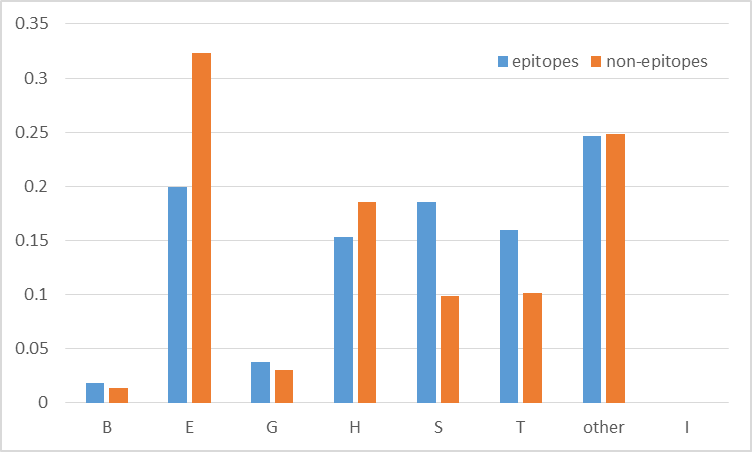


**S1 Fig. Secondary structure of epitopes and non-epitopes. B: residue in isolated β-bridge ; E:extended strand, participates in β ladder; G:3-helix;H:α-helix;S:bend;T:hydrogen bonded turn; I:5 helix.**
