# Supplementary material for: Analysis of Conformational B-Cell Epitopes in the Antibody-Antigen Complex Using the Depth Function and the Convex Hull
Source: PLoS One. 2015 Aug 5;10(8):e0134835. doi: 10.1371/journal.pone.0134835 (PMC4526569; doi:10.1371/journal.pone.0134835)
Supplement: S2 Fig — HSEA:Half-Sphere Exposure using information only about the Cα position. (DOC) [file pone.0134835.s002.doc]

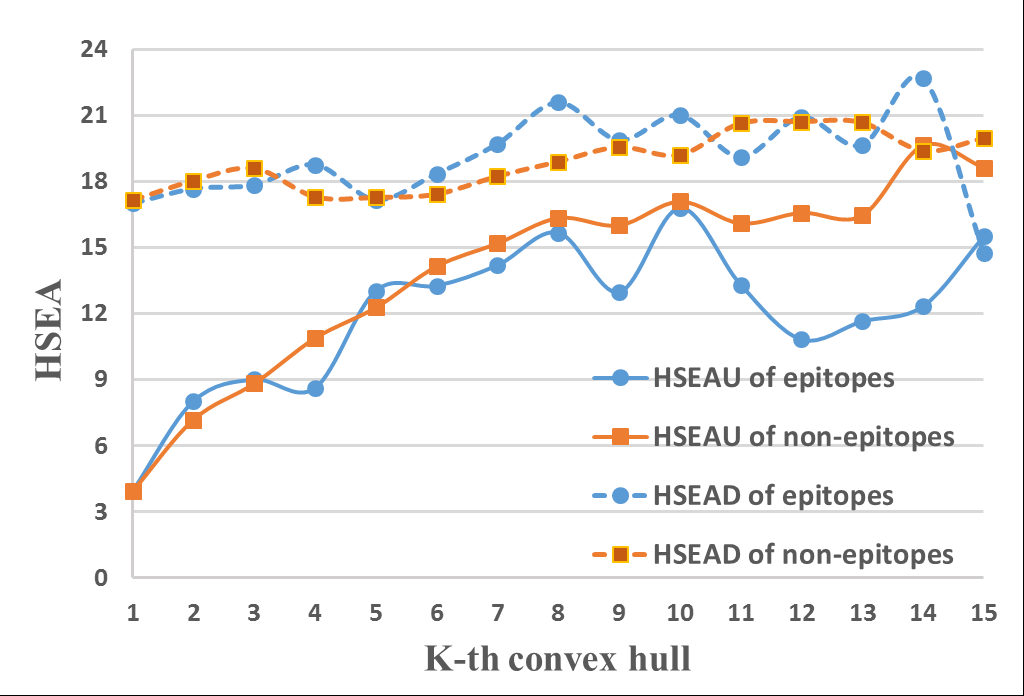


**S2 Fig. HSEA depth functions according to k-th convex hull layers CH_k_ (k=1, 2,…, 15). HSEA:Half-Sphere Exposure using information only about C_α_ position.**
