# Supplementary material for: Analysis of Conformational B-Cell Epitopes in the Antibody-Antigen Complex Using the Depth Function and the Convex Hull
Source: PLoS One. 2015 Aug 5;10(8):e0134835. doi: 10.1371/journal.pone.0134835 (PMC4526569; doi:10.1371/journal.pone.0134835)
Supplement: S4 Fig — There is no significantly difference between epitopes and non-epitopes in the top 8 convex hull layers (p-values are 0.82, 0.08, 0.60, 0.37, 0.53, 0.42, 0.24 and 0.64 using Wilcoxon rank sum test with two sides). (DOC) [file pone.0134835.s004.doc]

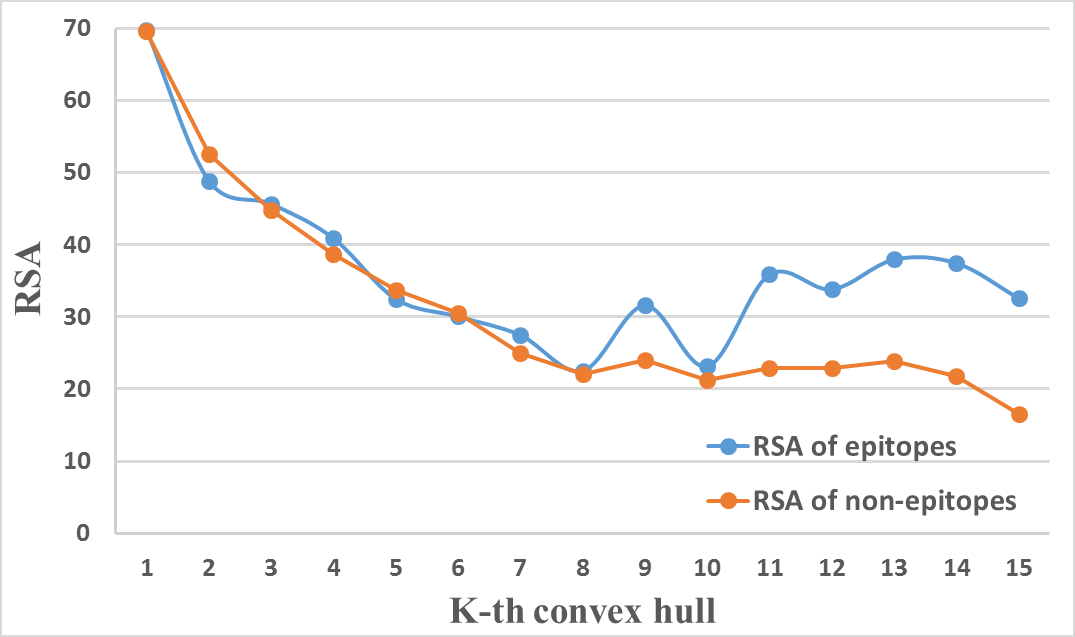


**S4 Fig. RSA according to k-th convex hull layers CH_k_ (k=1, 2,…, 15). There is no significantly difference between epitopes and non-epitopes in the top 8 convex hull layers (p values are 0.82, 0.08, 0.60, 0.37, 0.53, 0.42, 0.24 and 0.64 using Wilcoxon rank sum test with two sides).**
